# Supplementary material for: Active Nanointerfaces Based on Enzyme Carbonic Anhydrase and Metal–Organic Framework for Carbon Dioxide Reduction
Source: Nanomaterials (Basel). 2021 Apr 15;11(4):1008. doi: 10.3390/nano11041008 (PMC8071118; doi:10.3390/nano11041008)
Supplement: Supplementary file 1 [file nanomaterials-11-01008-s001.zip › nanomaterials-1165155-supplementary.pdf]

nanomaterials

Supporting Information

Active Nanointerfaces Based on Enzyme Carbonic Anhydrase and Metal–Organic Framework for Carbon Dioxide Reduction

Qian Liu, Xinwei Bai, Huy Pham, Jianli Hu and Cerasela Zoica Dinu *

Department of Chemical and Biomedical Engineering, West Virginia University, Morgantown, WV 26506, USA; ql0009@mix.wvu.edu (Q.L.); xb0001@mix.wvu.edu (X.B.); hgpham@mix.wvu.edu (H.P.); john.hu@mail.wvu.edu (J.H.)
* Correspondence: Cerasela-Zoica.Dinu@mail.wvu.edu; Tel.: +1-304-293-9338

Schemes, Figures and Tables

"*In house*" platform built to evaluate changes in the CO_2 concentration as resulted upon gas adsorption at the CA-membrane interface. The unit integrated a micro GC, a mass flow controller and a membrane module; the micro GC was used to monitor the concentration of CO_2 gas and any differences in the gas concentration in the inlet and outlet of the pipeline respectively. The mass flow reactor was used to control the gas flow, while the membrane module was used to store the CA-based interface where the CO_2 adsorption was to occur.

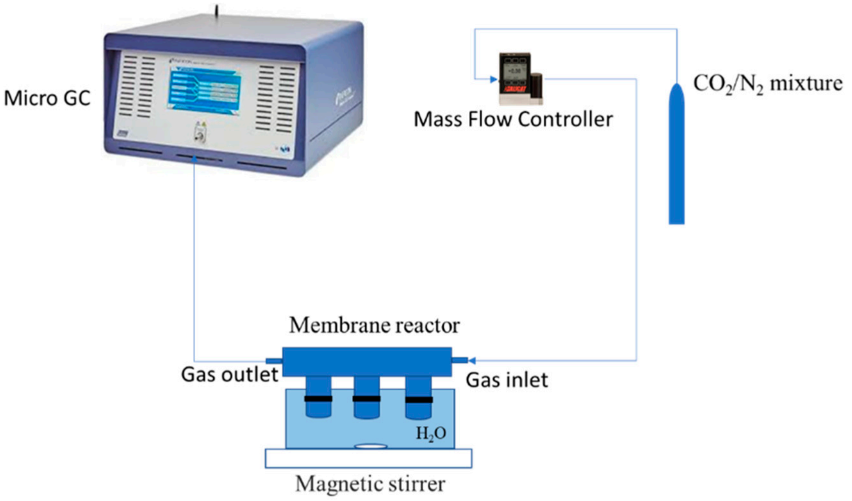

Scheme 1. Schematic illustration of "*in house*" platform used to evaluate changes in CO_2 concentration at the enzymatic membrane interface.

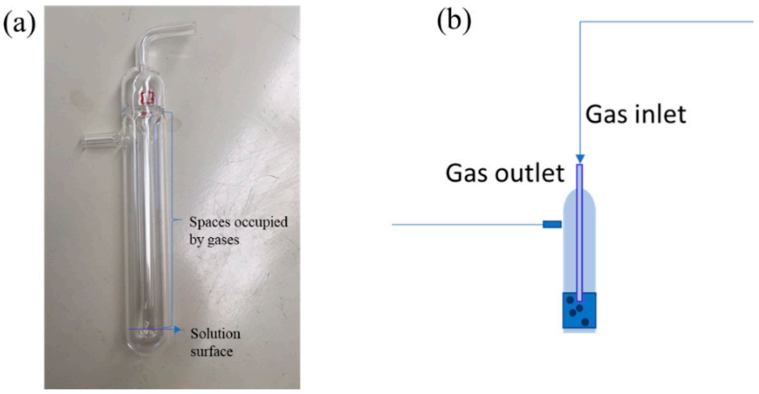

Scheme S2. Schematic illustration of the reactor used for monitoring changes in CO_2 concentration upon gas interaction with a solution containing free CA.

AFM in AC mode was used to evaluate changes in samples height profiles as resulted from sample functionalization. Results showed significant height changes upon FDCA binding onto the filter's surface.

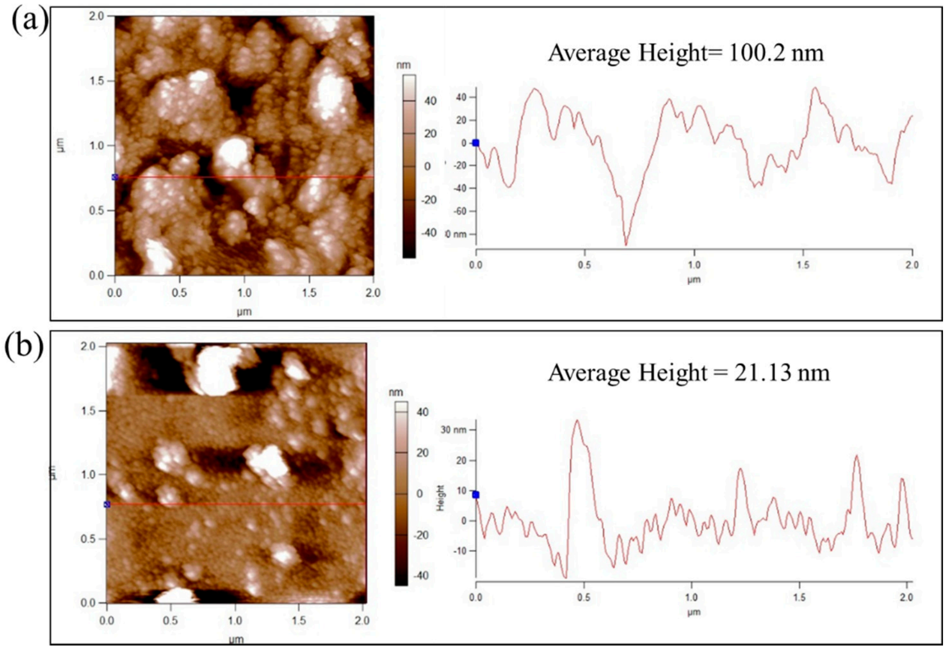

Figure S1. AFM images of FDCA/Al_2O_3 functionalized filter (a) and Al_2O_3 filter (b) with their corresponding average height profile.

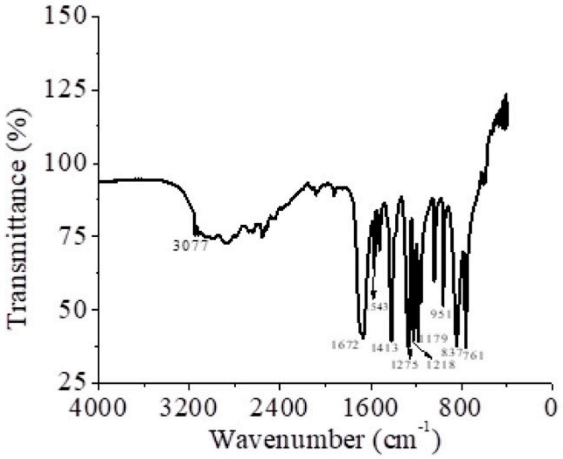

Figure S2. FTIR spectra of FDCA linker.

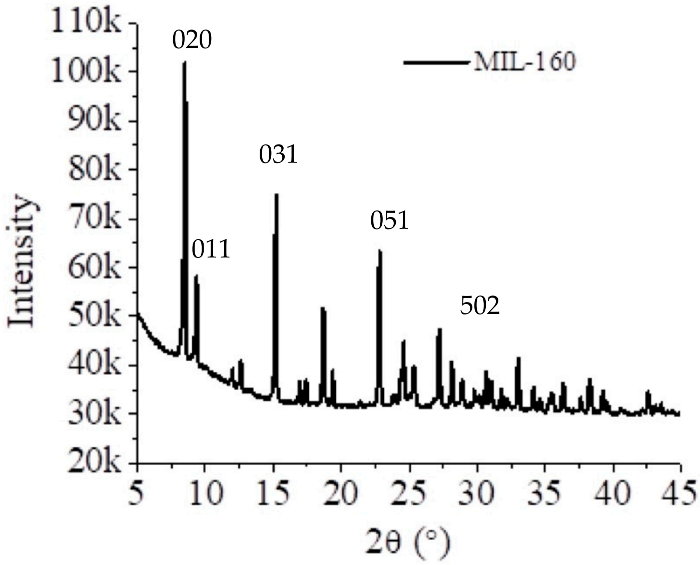

Figure 3. XRD spectra of the MOF.

Table 1. Elemental composition of control and FDCA/Al_2O_3 functionalized filter.

Sample	Element	Atomic concentration (%)
Al_2O_3	C K	8.59 ± 0.36
	O K	37.48 ± 0.19
	Al K	53.93 ± 0.21
FDCA/Al_2O_3	C K	12.45 ± 0.08
	O K	44.45 ± 0.11
	Al K	43.11 ± 0.09

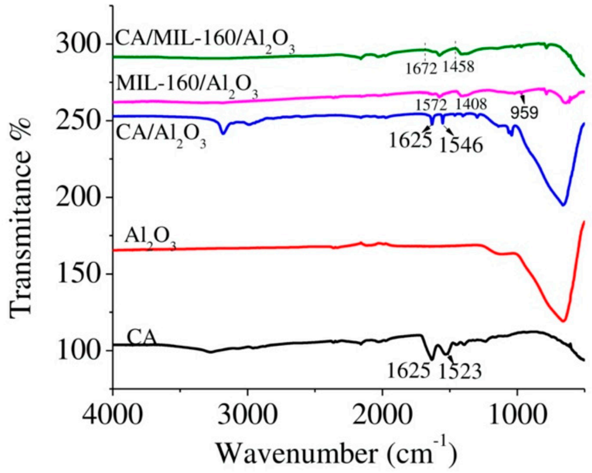

Figure 4. FTIR analysis of membranes and controls.

Equations S1–3 supporting previously established mechanism for CO_2 transformation by CA

$$CO_2 + ZnOH^- (E_H) \rightleftharpoons ZnHCO_3^- (ES)$$

$$H_2O + ZnHCO_3^- (ES) \rightleftharpoons HCO_3^- + ZnH_2O (E_w)$$

$$B + ZnH_2O (E_w) \rightleftharpoons BH^+ + ZnOH^- (E_H)$$

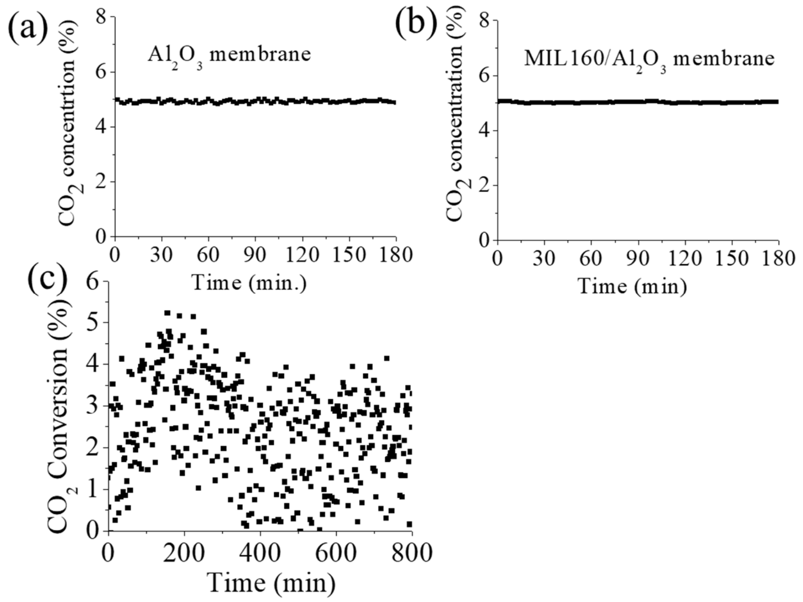

Figure 5. CO_2 adsorption at the Al_2O_3 filter (a), MIL-160/Al_2O_3 hybrid (b) and free CA in deionized water (c) interfaces.

Table S2. Comparison of the CO_2 hydration efficiency of the user synthesized CA/MIL-160/Al_2O_3 relative to other reports.

Membrane	CO_2 hydration rate, mol $s^{-1}m^{-2}$	Effective Membrane area, m^2	Solvent	Reference
**CA/MIL-160/Al_2O_3**	1.8×10^{-2}	1.2×10^{-4}	Water	This work
**CA-FTCS-CNTs-PVDF flat sheet membrane**	2.1×10^{-4}	-----------	Water	[3]
**CA-PDA-PEI--PVDF hollow fiber membrane**	2.5×10^{-3}	6.4×10^{-4}	Water	[4]
